# Marginal Level Dystrophin Expression Improves Clinical Outcome in a Strain of Dystrophin/Utrophin Double Knockout Mice

**DOI:** 10.1371/journal.pone.0015286

**Published:** 2010-12-20

**Authors:** Dejia Li, Yongping Yue, Dongsheng Duan

**Affiliations:** Department of Molecular Microbiology and Immunology, University of Missouri, Columbia, Missouri, United States of America; Johns Hopkins, United States of America

## Abstract

Inactivation of all utrophin isoforms in dystrophin-deficient mdx mice results in a strain of utrophin knockout mdx (uko/mdx) mice. Uko/mdx mice display severe clinical symptoms and die prematurely as in Duchenne muscular dystrophy (DMD) patients. Here we tested the hypothesis that marginal level dystrophin expression may improve the clinical outcome of uko/mdx mice. It is well established that mdx3cv (3cv) mice express a near-full length dystrophin protein at ∼5% of the normal level. We crossed utrophin-null mutation to the 3cv background. The resulting uko/3cv mice expressed the same level of dystrophin as 3cv mice but utrophin expression was completely eliminated. Surprisingly, uko/3cv mice showed a much milder phenotype. Compared to uko/mdx mice, uko/3cv mice had significantly higher body weight and stronger specific muscle force. Most importantly, uko/3cv outlived uko/mdx mice by several folds. Our results suggest that a threshold level dystrophin expression may provide vital clinical support in a severely affected DMD mouse model. This finding may hold clinical implications in developing novel DMD therapies.

## Introduction

Duchenne muscular dystrophy (DMD) is a prevalent lethal muscle disease caused by dystrophin gene mutation [1]. Affected boys often fail to reach development milestones that are related to muscle function (such as crawling and walking). Most patients are wheelchair-bound at early teenage and die prematurely in early adulthood. Restoring dystrophin expression may lead to a cure. However, it is a technically rather daunting, if not impossible, task to recover dystrophin expression to the wild type level in every muscle in the body. For this reason, it is important to determine whether a marginal level expression can improve life quality and prolong lifespan.

In sharp contrast to human patients, dystrophin-deficient mdx mice display very mild symptoms. Despite clear histological evidence of muscle damage, young adult mdx mice do not show muscle wasting and they maintain a close-to-normal daily activity. Further, the lifespan of mdx mice is only moderately reduced [2], [3]. One mechanism underlying reduced clinical presentation of mdx mice is the up-regulation of utrophin, a ubiquitously expressed dystrophin paralogue. Crossing mdx mice to the utrophin-null background results in utrophin knockout mdx (uko/mdx) mice. These double deficient mice present severe clinical manifestations of muscular dystrophy [4], [5]. They show growth delay, muscle wasting, reduced mobility and a significantly shortened lifespan [2], [4], [5], [6].

Mdx3cv (3cv) mice carry a point mutation that abolishes all wild type dystrophin transcripts (Figure S1) [7]. Interestingly, it results in an exon 65/66 truncated transcript (Figure S1) [7]. This alternative transcript translates into a near-full-length dystrophin protein at approximately 5% of the normal level [7], [8]. Transgenic study suggests that exon 65/66-deleted dystrophin is localized at the sarcolemma and it is capable of restoring syntrophin expression. Nevertheless, this slightly truncated protein fails to anchor β-dystroglycan to the membrane [9]. Additional studies suggest that low-level expression of this near-full-length dystrophin enhances muscle function in 3cv mice although it does not reduce histopathology [8], [10]. It is currently unclear whether such marginal expression can improve the clinical outcome in a severe DMD model. To address this issue, we crossed the 3cv mutation to a uko strain in which all utrophin isoforms are eliminated [5]. We then analyzed the clinical phenotype of the resulting utrophin knockout 3cv (uko/3cv) mice. Similar to uko/mdx mice, utrophin expression and full-length dystrophin expression were eliminated in uko/3cv mice. However, uko/3cv mice expressed a slightly shortened dystrophin protein at the same level as that of 3cv mice. Surprisingly, we observed dramatic clinical improvement. Compared to uko/mdx mice, uko/3cv mice showed significantly higher body weight, better muscle force and longer lifespan. These findings suggest that marginal level dystrophin expression may hold clinical significance under certain conditions.

## Materials and Methods

### Animals

**Figure 1:**
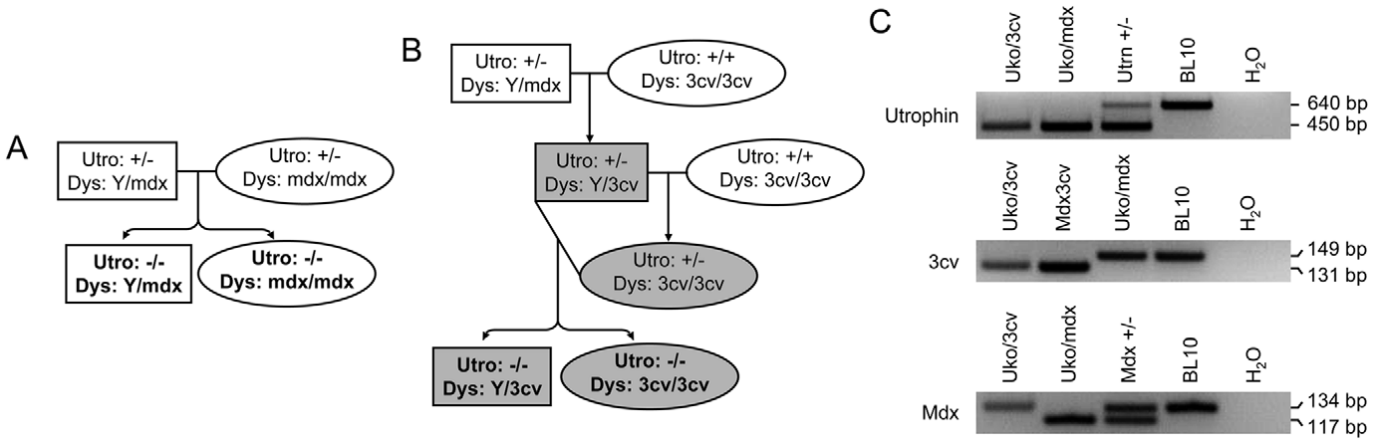
Generation of uko/mdx and uko/3cv mice. **A,** Outline of the breeding scheme to generate uko/mdx mice; **B,** Outline of the breeding scheme to generate uko/3cv mice. Rectangle, male mice; oval, female mice; white background, pure genetic background; gray background, mixed genetic background; bolded letters, experimental mice. **C,** Representative genotyping photomicrographs. Top panel, utrophin PCR. Wild type yields a 640 bp band. Knockout yields a 450 bp band. Heterozygous yields both bands. Middle panel, mdx3cv PCR. Wild type yields a 149 bp band. Mdx3cv yields a 131 bp band. Bottom panel, mdx PCR. Wild type yields a 134 bp band. Mdx yields a 117 bp band. Heterozygous yields both bands. Utrn +/−, utrophin heterozygous; Mdx +/−, heterozygous mdx mice; H_2_O, negative control (without DNA template).

All animal experiments were approved by the Animal Care and Use Committee of the University of Missouri and were in accordance with NIH guidelines. C57Bl/10Snj (BL10), C57BL/10ScSn-*Dmd* ^mdx^/J (mdx), and B6Ros.Cg-*Dmd^mdx-3Cv^*/J (3cv) mice were purchased from The Jackson Laboratory (Bar Harbor, ME). The original utrophin heterozygous mdx (utrn^+/−^/mdx) breeders were gifts of Dr. Mark Grady at Washington University [5], [11]. Experimental uko/mdx and uko/3cv mice were generated by in house breeding (Figure 1A and B).

### Genotyping

Experimental mice were genotyped for utrophin knockout, 3cv mutation, and mdx mutation using strain-specific three-primer PCR protocols as we reported [11], [12]. Wild type utrophin yields a 640 bp diagnostic band. The diagnostic utrophin knockout band is at 450 bp. For 3cv genotyping, the wild type allele yields a 149 bp band and the mutant allele yields a 131 bp band. For mdx genotyping, the wild type allele yields a 134 bp band and the mutant allele yields a 117 bp band.

### Western blot

Western blot was performed according to a previously published protocol [8]. Whole muscle lysate was obtained from freshly isolated tibialis anterior muscle. Dystrophin was detected with the Mandra1 antibody (1∶100; clone 7A10, IgG1; a gift from Dr. Glenn Morris, The Robert Jones and Agnes Hunt Orthopaedic Hospital, UK) [13]. Utrophin was detected with a mouse monoclonal antibody against utrophin amino acid residues 768-874 (#610896, 1∶250; clone 55, IgG1; BD Biosciences, San Diego, CA). For the loading control, membrane was probed with an anti-α-tubulin antibody (1∶3,000; clone B-5-1-2; Sigma, St Louis, MO). The relative level of dystrophin expression was quantified with the Image J software (version 1.36b). For each blot, dystrophin in BL10 muscle was arbitrarily defined as 100%.

### Immunofluorescence staining

Dystrophin immunofluorescence staining was performed according to a published protocol [2], [8]. The primary antibody was Dys-2 (1∶30; Vector Laboratories, Burlingame, CA). The secondary antibody was an Alexa Fluor 594 conjugated F(ab')_2_ fragment of goat anti-mouse IgG (H+L) (1∶100; Invitrogen, Carlsbad, CA).

### In vitro evaluation of the EDL muscle force

The absolute twitch and tetanic (80, 120, and 150 Hz) forces of the EDL muscle were measured in vitro at 30°C using a 300B dual-mode servomotor transducer and a DMC/DMA software (Aurora Scientific, Inc., Aurora, Ontario, Canada) as we published before [8], [11], [14], [15]. Muscle cross-sectional area (CSA) was calculated according to the following equation, CSA  =  (muscle mass)/(0.44 x Lo x muscle density). 0.44 represents the ratio of fiber length to optimal muscle length (Lf/Lo) for the EDL muscle. Muscle density is 1.06 g/cm^3^. The specific force (kN/m^2^) was calculated by normalizing the absolute muscle force with the CSA. After tetanic force measurement, the muscle was rested for 10 min and then subjected to five rounds of eccentric contraction injury according to our previously published protocol [8], [11], [14], [15]. The percentage of force drop following each round of eccentric contraction was recorded.

### Statistical analysis

Data are presented as mean ± standard error of mean. Statistical analysis for body weight and EDL muscle contractility was performed with the SPSS software (SPSS, Chicago, IL). Statistical significance was determined by t test. Statistical analysis for survival was performed with the Prism 4 software (GraphPad, San Diego, CA). Statistical significance was determined by the Mantel-Cox log-rank test. The Bonferroni corrected threshold was used to set the significance level. Difference was considered significant when *P*<0.05.

## Results

### Generation of uko/3cv mice

Experimental uko/mdx mice were generated by crossing utrophin heterozygous mdx mice (Figure 1A) [5]. Experimental uko/3cv mice were generated through three rounds of breeding (Figure 1B). We first generated male utrophin heterozygous 3cv mice by crossing male utrophin heterozygous mdx mice with female 3cv mice. We then generated female utrophin heterozygous 3cv mice by crossing male utrophin heterozygous 3cv mice with female 3cv mice. Finally utrophin heterozygous 3cv mice were crossed to generate uko/3cv mice (Figure 1B). The genotype of all experimental mice was verified by PCR (Figure 1C) [11], [12].

**Figure 2:**
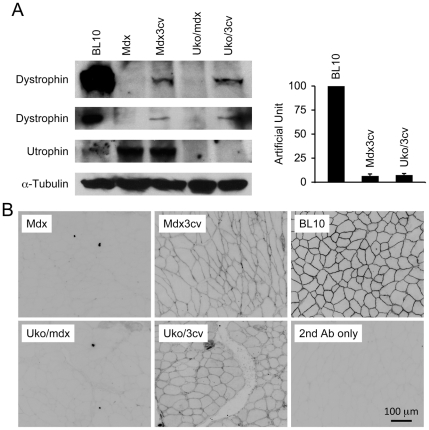
Dystrophin expression in uko/mdx and uko/3cv mice. **A,** Left panel, Representative western blots from skeletal muscle of 2.5-m-old mice. Top two rows show representative dystrophin expression from two independent experiments. The third row shows representative utrophin expression. α-Tubulin western blot (the bottom row) serves as the loading control. Right panel, Densitometry quantification of dystrophin expression. N = 4 for BL10, N = 3 for uko/mdx and N = 4 for uko/3cv. **B,** Representative dystrophin immunofluorescence staining. Dystrophin antibody was not applied to the slide in the 2nd antibody only photomicrograph.

Dystrophin and utrophin expression was confirmed by western blot (Figure 2A). In uko/mdx mice, dystrophin and utrophin were essentially undetectable (Figure 2A). In uko/3cv mice, utrophin was completely eliminated while a near-full length dystrophin was seen at a level comparable to that of 3cv mice (Figure 2A) [7], [8]. Marginal level dystrophin expression in 3cv and uko/3cv mice was further illustrated by immunofluorescence staining (Figure 2B).

### Body weight and life span were significantly improved in uko/3cv mice

**Figure 3:**
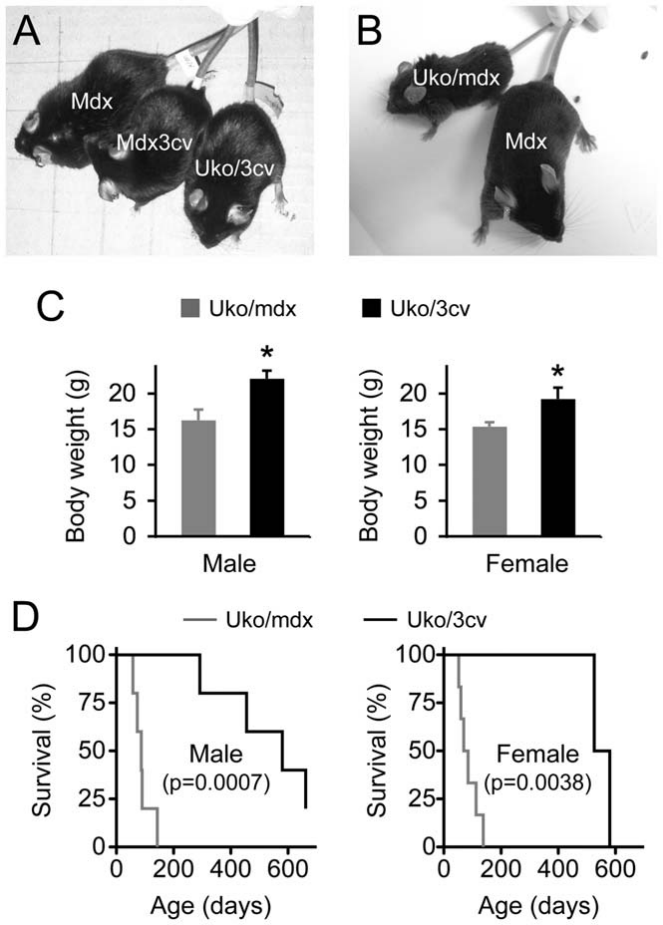
Body weight and survival of uko/mdx and uko/3cv mice. **A,** Representative photomicrographs of 8-m-old male mdx, mdx3cv and uko/3cv mice. **B,** Representative photomicrographs of 2-m-old male mdx and uko/mdx mice. **C,** Body weight of 2.5-m-old uko/mdx (N = 11 for male and N = 9 for female) and aged-matched uko/3cv (N = 6 for male and N = 3 for female) mice. Asterisk, significantly different from the other group (p<0.02). **D,** Kaplan–Meier survival curves for uko/mdx (N = 5 for male and N = 6 for female) and uko/3cv (N = 6 for male and N = 4 for female) mice.

At 8 months of age, mdx, 3cv and uko/3cv were essentially indistinguishable on visual examination (Figure 3A). There was also no apparent activity deficiency in 8-m-old uko/3cv mice (Video S1). In contrast, uko/mdx mice appeared clearly emaciated at 2 months of age (Figure 3B). In age-matched (2.5-m-old) mice, the body weight of uko/3cv mice was significantly higher than that of uko/mdx mice irrespective of the gender (Figure 3C).

All uko/mdx mice die within four months after birth [2], [4], [5], [6]. Surprisingly, all uko/3cv mice were alive at this age (Figure 3D). The median survival age of male and female uko/3cv mice reached 580 and 553 days, respectively.

### The specific force of the uko/3cv EDL muscle significantly outperformed that of uko/mdx mice

**Figure 4:**
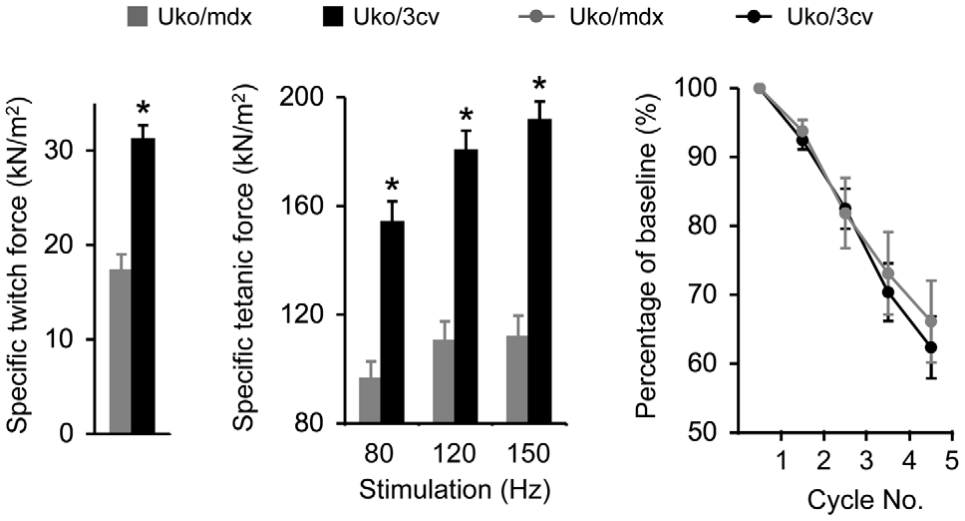
Characterization of the EDL muscle contractile profiles in 2.5-m-old male uko/mdx and uko/3cv mice. Left panel, Specific twitch force. Middle panel, Specific tetanic forces. Right panel, Relative force decline following five rounds of eccentric contraction. Sample size, N = 8 for uko/mdx, N = 6 for uko/3cv. Asterisk, significantly different from the other group (p<0.0001).

To study the influence of marginal level dystrophin expression on muscle contractility, we measured the anatomic property, twitch/tetanic force and eccentric contraction profile of the EDL muscle in 2.5-m-old male mice. Compared to that of uko/mdx mice, muscle weight, optimal length and cross-sectional area were significantly increased in uko/3cv mice (Table 1). Uko/3cv also showed significantly higher specific twitch and tetanic forces. Interestingly, both strains showed a similar eccentric contraction profile (Figure 4).

**Table 1:** Anatomic features of the EDL muscle in 2.5-m-old mice.

Strain	N	Weight (mg)	Lo (mm)	CSA (mm^2^)
uko/mdx	8	6.39±0.99	10.67±0.53	1.24±0.14
uko/3cv	6	9.52±0.31	12.51±0.17	1.64±0.07
p value		0.021	0.013	0.04

## Discussion

Tremendous progress has been achieved over the last few years in developing novel pharmacological and genetic therapies for treating DMD. Many approaches (such as stop codon read-through and exon skipping) aim at expressing a functional dystrophin molecule [16], [17]. It is unlikely that such attempts will completely restore dystrophin expression to the wild type level. To determine whether marginal level dystrophin expression is clinically relevant, we compared body weight, life span and EDL muscle contractility between uko/mdx and uko/3cv mice. These mice carry point mutations in the dystrophin gene and null mutation of all utrophin genes. As a result, utrophin and full-length dystrophin expression is abolished.

Uko/mdx mice are well-established clinical DMD models [4], [5]. They experience significant growth retardation and die prematurely (Figure 3) [2], [4], [5], [6]. 3cv mice expressed a near full-length dystrophin protein at approximately 5% of the normal level (Figure 2) [2], [7]. Uko/3cv mice yielded similar expression (Figure 2). Surprisingly, uko/3cv mice developed a much milder phenotype. Their body weight was significantly higher at two and half months of age and their daily activity appeared minimally impaired at eight months of age (Figure 3C, Video S1). Further, the EDL muscle of uko/3cv mice showed significantly enhanced specific twitch and tetanic forces (Figure 4). Remarkably, we observed a significantly increased life span in uko/3cv mice. The median survival reached ∼1.5 years (Figure 3D). The longest-lived uko/3cv mouse did not succumb until 22-month-old.

A crucial issue in developing DMD therapy is to determine the minimal levels of dystrophin needed to mitigate the clinical manifestation. Previous transgenic studies suggest that dystrophin expression at 20% of the normal level is sufficient to ameliorate histological lesions and correct functional deficits in mdx skeletal muscle [18], [19]. We have also demonstrated that ∼5% dystrophin level significantly enhanced muscle force in mdx3cv mice although it failed to prevent sarcolemmal leakage and muscle necrosis [8]. Recently, a patient with 29% protein level dystrophin expression was found free of skeletal muscle disease at age 23 [20]. However, a female carrier who had 45% protein level dystrophin expression on western blot was found with severe muscular dystrophy [21]. Interestingly, another female carrier with 30% level dystrophin expression showed a mild phenotype at age 63 [21]. Collectively, these results suggest that the clinical manifestation of DMD in a particular subject may be regulated by a number of factors. Our data here suggest that a threshold level of ∼5% dystrophin expression may meet the basic growth needs and prolong survival in a phenotypic DMD mouse model.

While the finding is encouraging, we would also like to point out several important limitations. First, the sample size is relatively small, especially for the life span study (Figure 3D, N = 6 for uko/3cv male and N = 4 for uko/3cv female). Unfortunately this is an inherent limitation of the study. Compared to other strains of dystrophin-deficient mice (such as mdx), 3cv mice show significantly reduced fertility due to abnormal spermatozoa [22]. We have also experienced great difficulties in the breeding. Nevertheless, all our uko/3cv mice outlived uko/mdx mice.

Second, uko/mdx mice were on the BL10 background [11]. However, uko/3cv mice were on a mixed genetic background of BL10 and C57Bl/6. It is possible the difference in the genetic background of the mouse strain may have also influenced the results. As a matter of fact, a series of recent publications have clearly demonstrated the dramatic leverage of various genetic modifiers on the dystrophic phenotype [23], [24], [25], [26]. Although not proved yet, variations in the dystrophin level and disease severity seen in DMD patients may also relate to the genetic modifiers [20], [21].

Third, Rafael et al. have previously reported an uko/3cv strain based on a different uko model [4]. Interestingly, their uko/3cv strain developed the same clinical phenotype as uko/mdx mice and none lived beyond 20 weeks of age [27]. This outcome is completely different from our observation (Figure 3). The exact reason of the discrepancy is currently not clear. One possibility may relate to the uko model used in the study. All utrophin isoforms are eliminated in the uko model we used [5]. However, expression of the smaller utrophin isoforms is not abolished in the uko strain used by Rafael et al [4]. Rafael et al indeed observed an up-regulation of 113 kD G-utrophin in the kidney in their uko/3cv mice [27], [28]. Forced expression of smaller dystrophin isoforms (such as Dp71 and Dp116) has been shown to worsen muscle disease of dystrophin-null mice by competing with up-regulated utrophin [29], [30], [31]. Future studies are needed to determine whether up-regulated smaller utrophin isoforms compete away the near-full length dystrophin protein from the sarcolemma in uko/3cv mice reported by Rafael et al.

Fourth, it should also be pointed out that our observation might relate to low-level Δ65/66 dystrophin expression during the embryonic stage. Several studies suggest that dystrophin deficiency causes fetal developmental abnormalities [32], [33], [34]. If this is the case, then the therapeutic implication of restoring low-level dystrophin expression later in life will be limited.

An interesting finding of the study is the seemingly different impact on the life span between male and female mice (Figure 3D). The median survival age of male uko/3cv mice was slightly longer than that of female. Male mice also appeared to die over a greater time span (Figure 3D). It is very likely that the difference is related to the small sample size of the current study. Future investigation with a larger sample size will help resolve the discrepancy. Nevertheless, it is worth to point out that gender may indeed play a role in dystrophin-deficient mice. A recent study by Bostick et al suggests that female mdx mice develop much severe cardiomyopathy than age-matched males [35].

In summary, we have demonstrated that minimal level dystrophin expression resulted in significant clinical improvement in a symptomatic mouse DMD model. Interestingly, a recent clinical trial with the read-through drug gentamicin also revealed a significant drop of the serum creatine kinase level despite only a marginal increase of dystrophin expression. Together, these results suggest that low-level dystrophin expression may still offer some clinical benefits for certain DMD patients [36].

## Supporting Information

Figure S1 **Schematic outline of RNA expression in mdx3cv mice. A,** Normal transcription/splicing yields an in-frame full-length transcript. **B** to **D,** Alternative transcripts in mdx3cv muscle. Only Δ65/66 transcript is in-frame. Red letter marks the mutation. (Note, this figure is based on the results published by Cox et al *Nature Genetics* 4:87-93, 1993).
(TIF)

Video S1 **8-m-old mdx, mdx3cv and uko/3cv male mice show similar activity.** The strain is marked by color tape on the tail. Gray, mdx; green, mdx3cv; red, uko/3cv.
(MPG)
