# Supplementary figures and images for: Marginal Level Dystrophin Expression Improves Clinical Outcome in a Strain of Dystrophin/Utrophin Double Knockout Mice
Source: PLoS One. 2010 Dec 20;5(12):e15286. doi: 10.1371/journal.pone.0015286 (PMC3004926; doi:10.1371/journal.pone.0015286)

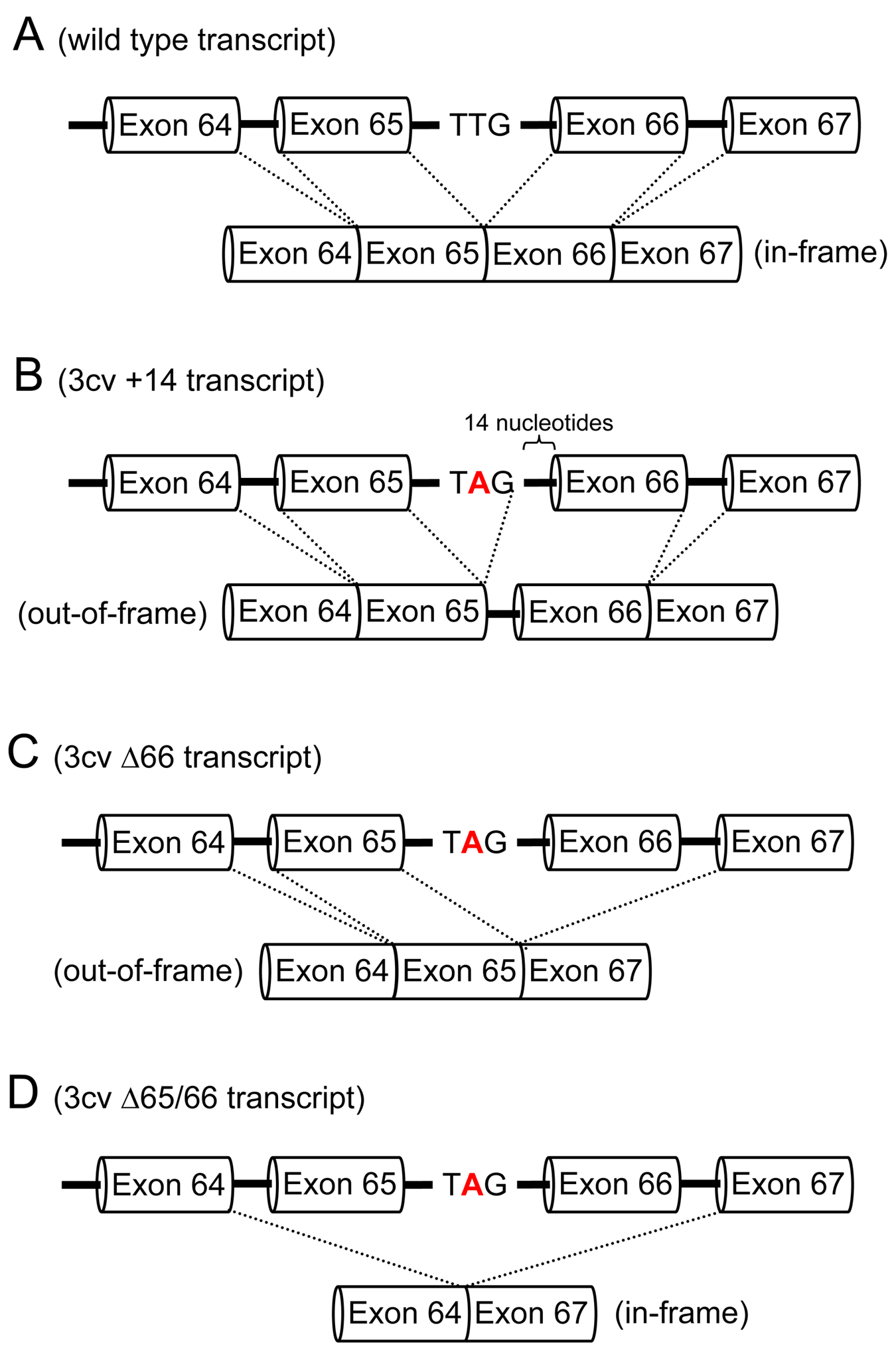

Supplement: Figure S1 — Schematic outline of RNA expression in mdx3cv mice. A, Normal transcription/splicing yields an in-frame full-length transcript. B to D, Alternative transcripts in mdx3cv muscle. Only Δ65/66 transcript is in-frame. Red letter marks the mutation. (Note, this figure is based on the results published by Cox et al Nature Genetics 4:87-93, 1993). (TIF) [file pone.0015286.s001.tif]
